# The Effectiveness of Rapamycin Combined with Eltrombopag in Murine Models of Immune-Mediated Bone Marrow Failure

**DOI:** 10.1155/2020/1798795

**Published:** 2020-10-17

**Authors:** Shaoxue Ding, Xiaowei Liang, Tian Zhang, Rong Fu

**Affiliations:** Department of Hematology, Tianjin Medical University General Hospital, Tianjin, China

## Abstract

Severe aplastic anemia (SAA) is a rare disease characterized by severe pancytopenia and bone marrow failure. Most patients with AA respond to immunosuppressive therapy (IST), usually as antithymocyte globulin (ATG) and cyclosporine (CsA), but some relapse on CsA withdrawal or require long-term administration of CsA to maintain blood counts. Recent research has found that rapamycin (Rapa) was an effective therapy in mouse models of immune-mediated bone marrow failure. However, it has not achieved a satisfactory effect in clinical application. At present, many studies have confirmed that eltrombopag (ELT) combined with IST can improve the curative effect of AA patients. Then, whether Rapa combined Elt in the treatment of AA will acquire better efficacy than a single drug application remains unclear. In this study, an immune attack-mediated AA mouse model was constructed by total body irradiation (TBI) and allo-lymphocyte infusion. In our study, we tested the efficacy of Rapa combined with Elt as a new treatment in mouse models of immune-mediated bone marrow failure. It showed that treatment with Rapa in combination Elt in the AA mouse model ameliorated pancytopenia and extended animal survival in a manner comparable to the standard dose of CsA and Rapa alone. However, there was no significant improvement effect on the number and function of NK cells and their subsets, mDCs, and CD4+/CD8+ ratio in AA mice after the therapy of Rapa combined with Elt compared with Rapa alone. Furthermore, the secretion of IL-10 of Tregs in AA mice increased significantly after the therapy of Rapa combined with Elt, but there was no significant difference in the number of Treg cells. We did not observe the difference in the curative effect of the Rapa group and CsA group, but for IL-10/Tregs ratio, the Rapa group was superior to the CsA group. And the IFN-r secretion of CD8+T cells in AA mice decreased significantly after the combination therapy of Rapa and Elt than Rapa alone. Compared with the AA group, the level of plasma IFN-*γ*, IL-2, and TNF-*α* decreased significantly (*P* < 0.05), but IL-10, IL-4, IL-5, and IL-1*β* increased significantly in the Rapa group (*P* < 0.05). As for IL-10, IL-12p70, IL-2, IL-6, KC/GRO, and TNF-*α*, the therapy of Rapa combined with Elt showed a more significant effect than Rapa alone in AA mice. To some extent, this study had shown a relatively better synergistic effect in murine models of immune-mediated bone marrow failure after the combination therapy of Rapa and Elt, which was a promising clinical utility in SAA treatment.

## 1. Introduction

Aplastic anemia (AA) is a rare and heterogeneous disorder. It is defined as pancytopenia with hypocellular bone marrow in the absence of an abnormal infiltrate or marrow fibrosis [1, 2]. For many years, the focus in the clinical development of the novel nontransplant therapies for severe aplastic anemia (SAA) has been on intensifying immunosuppressive therapy (IST), usually as antithymocyte globulin (ATG) and cyclosporine (CsA), which have dramatically changed the course of this illness, with the 10-year survival rate of patients now being about 70% [3]. However, about 30% of patients still have poor treatment response, and about 10% of responders relapse, while 16% of patients have treatment-related deaths due to drug-related side effects [4, 5].

Therefore, a novel immunosuppressive regimen to circumvent these problems is needed. Rapamycin is a macrocyclic antibiotic that blocks the multifunctional serine-threonine kinase, as an inhibitor of the mammalian target of rapamycin (mTOR) pathway that has been used to treat autoimmune diseases and to prevent rejection in solid organ and hematopoietic stem cell transplantation diseases [6, 7]. In recent years, Feng et al. [8] had reported that rapamycin effectively treated immune-mediated murine BM failure as compared to the standard-dose CsA, which showed a high efficacy in suppressing Th1 immune responses, eradicating pathogenic CD8+ T cells. However, previous clinical studies showed that the addition of rapamycin provided no significant benefit in outcome in SAA [9].

Eltrombopag (Elt), an oral thrombopoietin-receptor agonist, is the only thrombopoietin receptor (TPO-R) agonist approved for the treatment of SAA. Elt had received FDA approval for the treatment of SAA in recent years, based on phase I/II dose-escalation trial of a single agent Elt for patients failing one or more treatment cycles with ATG/CsA and the efficacy within clinical trials exploring its role in the first-line therapy in addition to standard IST [10–12]. Based on the above research, we tested the efficacy of Rapa combined with Elt as a new treatment in mouse models of immune-mediated bone marrow failure, whether a better curative effect can be achieved compared to the standard dose of CsA and Rapa alone.

In this article, an immune attack-mediated AA mouse model was constructed by total body irradiation (TBI) and allo-lymphocyte infusion [13]. We will discuss new insights into the possible efficacy of Rapa combined with Elt in BMF mouse, which may further improve the efficacy of AA patients.

## 2. Materials and Methods

### 2.1. Induction of Bone Marrow Failure Murine Model

Induction of immune-mediated BM failure was performed as previously reported [13, 14].CB6F1 mice (strain: 303, 8-10weeks old, SPF grade) and C57BL/6 mice (HFK Bioscience Co. Ltd., China) were used to build the BMF murine model. CB6F1 mice, except mice in the normal group, received a sublethal total body irradiation dose of 5.0 Gy from Model 143 Cesium-137 *γ*-irradiator four hours before the lymph node cell infusion. Inguinal, brachial, and axillary infusion of the lymph node cells were obtained from female C57BL/6 mice. Lymph cells (5 × 10^7^) were injected into CB6F1 mice through the vein angular for inducing BMF murine model. In most experiments, animals were bled and euthanized 17 days later to obtain tissue for histology and cytology. Some mice were maintained long-term to measure survival under normal animal care conditions. All animal experiments were approved by the Institutional Animal Care and Use Committee of the National Institute of State Scientific and Technological Commission.

### 2.2. Blood Counts

Blood was collected from the retro-orbital sinus into EDTA-added Eppendorf tubes. Complete blood counts (CBC) were performed in an automatic blood cell analyzer (MEK-722, Nihon Kohden).

### 2.3. Flow Cytometry (FCM)

Fresh drawn peripheral blood from mice was processed and stained with specific fluorochrome-conjugated monoclonal antibodies (mAbs) to perform FCM analysis. The mAbs for murine including FITC-CD27, APC-CD11b, PerCP-Vio770-TCR, PE-Vio770-NK1.1, PE-NKG2A, PE-NKG2D, FITC-CD8, PE-CD4, PE-IFN-*γ*, FITC-CD11c, PerCP-Vio770-MHC II, PE-CD80, PE-CD86, PerCP-CD25, PE-FOXP3, APC-IL-10, and APC-CD152 were purchased from BD Pharmingen (Franklin Lakes, NJ, USA). NK cells were identified as NK1.1+TCR*β*-cells in mice. Intracellular IFN-*γ* and IL-10 were detected before the cells were incubated with Brefeldin A (1 *μ*g/ml) (R&D Systems) to inhibit cytokine exocytosis at 37°C for 6 h.

### 2.4. Administration of Immunosuppressive Agents and/or Eltrombopag to AA Model Mice

The treatment was initiated after lymph node cell infusion and lasted for 10 days. Mice were randomly divided into 6 groups: AA group, mice were fed the control diet and orally administered sterile saline; TBI group, mice were fed the same as the normal group; CsA (Zhongmei Huadong Pharmaceutical Co. LTD, Hangzhou, China) group, mice were fed the same control diet and intraperitoneal injection daily administered 50 *μ*g/g/d for 10 days; rapamycin (Rapa) (Solarbio, China) group, mice were fed the same control diet and intraperitoneal injection daily administered 2 *μ*g/g/d for 10 days; Elt group, mice were fed the same control diet and intraperitoneal injection daily administered Elt (20 *μ*g/g/d) for 10 days; and Rapa+Elt group, mice were fed the same control diet and intraperitoneal injection daily administered Rapa and Elt (20 *μ*g/g/d) for 10 days. The mice were sacrificed on the 17th day after treatment. Mice were anesthetized by isoflurane anesthesia (2-3% isoflurane with oxygen supply). Peripheral blood samples were collected by removing the eyeballs, and BMCs were obtained by the femoral cavity flushing.

### 2.5. Detecting the Expression of Each Factor in the Plasma of Mice in Each Group by MSD

SULFO-TAG_TM_ marker was used in the MSD electrochemiluminescence detection technology. First, the plasma samples of mice were collected and stored at -80°C. Then, we tested the level of plasma cytokines in the mice according to the operation instructions of MSD. The detection steps are as follows: (1) prepare the standard: add 1 ml of diluent to the standard, shake well and place at room temperature for 15-20 min, as the highest concentration point, and dilute it 4 times in turn, 7 standards and 1 blank. (2) Prepare the antibody diluent for detection: suck 60 *μ*l of the antibody for detection, and supplement 3 ml with diluent; prepare 1× PBS (0.05% Tween-20) as cleaning solution and configure 2× reading liquid. (3) Clean MSD board 3 times with 150 *μ*l cleaning solution each time. (4) Add 50 *μ*l of sample and standard into each hole, seal with sealing film, and shake at room temperature for 2 hours. Then, clean the MSD board 3 times with 150 *μ*l cleaning solution each time. (5) Add 25 *μ*l prepared antibody to each hole, seal with sealing film, and shake at room temperature for 2 hours. Then, clean the MSD board 3 times with 150 *μ*l cleaning solution each time. (6) Add 150 *μ*l of configured reading liquid into each hole then measure the plates using an MSD Sector Imager 6000 instrument. The data were analyzed using the SoftMax Pro 4.6 Enterprise Edition.

### 2.6. Statistical Analysis

Differences in quantitative parameters between groups were assessed using the *t* test or one-way ANOVA or nonparametric test. Differences in qualitative data were compared using *χ*^2^ test. The quantitative data were expressed as mean ± SD. The SPSS 19.0 software (SPSS Inc., Chicago, IL) was used for statistical analyses. All *P* values represented were 2-sided, with *P* < 0.05 indicating statistical significance.

## 3. Results

### 3.1. Rapamycin Combined with Eltrombopag Ameliorates Pancytopenia in AA Mice

**Figure 1:**
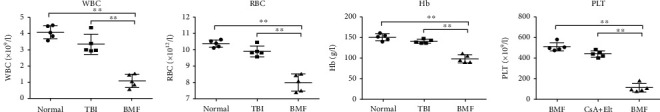
The complete blood counts of the AA mice were lower compared with the NC and TBI groups. ^∗^*P* < 0.05; ^∗∗^*P* < 0.01.

**Figure 2:**
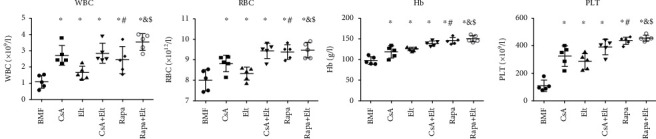
Improve the complete blood counts of the AA mice after rapamycin and eltrombopag treatment. ^∗^Compared with the AA group (*P* < 0.05). ^#^Compared with the CsA group (*P* > 0.05). ^&^Compared with the Rapa group (*P* < 0.05). ^$^Compared with the Rapa group (*P* < 0.05).

The BMF mouse model was successfully established as the pancytopenia was achieved in our study. The counts of WBC, RBC, Hb, and PLT in the peripheral blood of the AA mice were lower compared with the NC and TBI groups (Table 1, Figure 1) (*P* < 0.01). Compared with the AA group, the whole blood cell count of the CsA group, Rapa group, and Rapa+Elt group increased significantly, but the blood cell count improvement of the Elt group was significantly weaker than that of the other groups (Table 1, Figure 2) (*P* < 0.05). Then, compared with the CsA or Rapa single treatment group, there is an increasing trend in the whole blood cell count in the Rapa combined with Elt group, and the WBC increased most obviously (*P* < 0.05). But there was no significant difference between the CsA and Rapa groups (*P* > 0.05).

### 3.2. Efficacy of Rapamycin Combined with Eltrombopag on the Numbers of NK Cells

**Figure 3:**
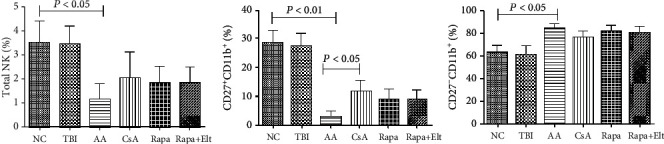
Efficacy of rapamycin combined with eltrombopag on numbers of NK cells. As for the CsA group, Rapa group, and Rapa+Elt group, the number of total NK cells and CD27+CD11b+NK cells were higher than the AA group, and CD27-CD11b+ NK cells were lower than the AA group. However, the number of CD27+CD11b+ NK cells increased only in the CsA group and was statistically significant (*P* < 0.05). There was no obvious difference in the numbers of NK cells, and their subsets were noted in the Rapa group, CsA group, and Rapa+Elt group.

NK cell is previously reported as a key immune regulatory cell. To facilitate the study of the effect of Rapa or Rapa combined with Elt on NK cells in AA mice, we generated immune-mediated AA mice that harbor a clinical manifestation of pancytopenia. As we expected, the number of total NK cells was significantly decreased in the AA group compared with the NC group (*P* < 0.05). In contrast, the number of NK cells in the CsA group, Rapa group, and Rapa+Elt group are slightly increasing, but not statistically significant. We next made a side-by-side comparison of the ration of peripheral regulatory NK (CD27+CD11b+) cells in different groups. The number of regulatory NK cells was observed notably decreased in the AA group compared with the NC group. As for the CsA group, Rapa group, and Rapa+Elt group, all showed an increased ratio of the peripheral NK cells compared with the AA group, but only the CsA group was statistically significant (*P* < 0.05). However, there is no notable difference in the ratio of regulatory NK cells between the Rapa group and Rapa+Elt group. To determine the effect of Rapa+Elt on the peripheral functional NK (CD27-CD11b+) cells, we performed flow cytometry. In the AA group, CD27-CD11b+NK cells were obviously increased compared with the NC group and TBI group, while its absolute number dropped (mainly regulatory NK cells). There is no notable change between the AA group and CsA group, as well as the Rapa group and Rapa+Elt group. There was no obvious difference in the numbers of NK cells, and their subsets were noted in the Rapa group and CsA group. Finally, no obvious difference in the numbers of NK cells and their subsets was noted in the Rapa group and Rapa+Elt group (Figure 3).

### 3.3. Efficacy of Rapamycin Combined with Eltrombopag on NK Functional Molecules

**Figure 4:**
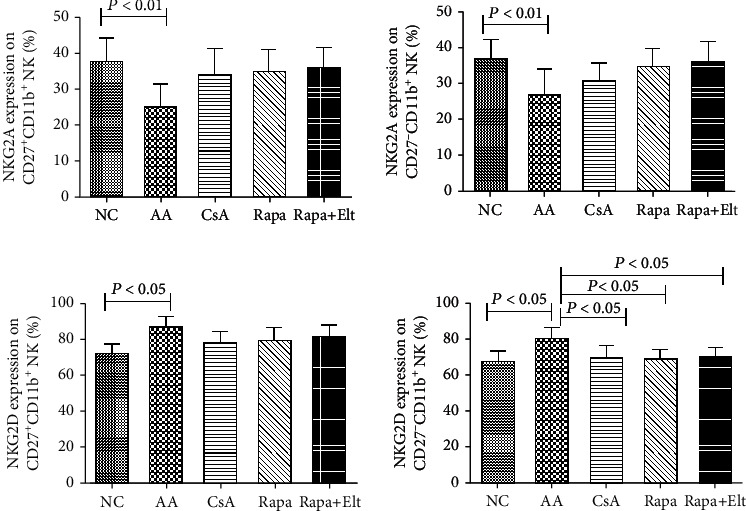
Efficacy of rapamycin combined with eltrombopag on NK functional molecules. The expression of NKG2A was significantly downregulated and NKG2D upregulated in the AA group compared with the NC group (*P* < 0.05). The expression of NKG2A on peripheral regulatory and functional NK cells was upregulated in the CsA group, Rapa group, and Rapa+Elt group (*P* > 0.05). The expression of NKG2D on the peripheral regulatory and functional NK cells was downregulated in the CsA, Rapa, and Rapa+Elt groups compared with the AA group, but there is statistical significance only for functional NK cells (*P* < 0.05). Moreover, there is no notable difference in the NKG2A and NKG2D expression between the Rapa group and Rapa+Elt group. There was no significant difference between the effect of Rapa and CsA on the function of NK cells.

To characterized the effect of Rapa combined with Elt on NK cell functional molecules, we used flow cytometry and measured the fluorescence density. In peripheral regulatory and functional NK cells, NKG2A was significantly downregulated in the AA group compared with the NC group (*P* < 0.01). The expression of NKG2A on the peripheral regulatory and functional NK cells was upregulated in the CsA group, Rapa group, and Rapa+Elt group compared with the AA group, but there is no statistical significance (*P* > 0.05). Moreover, there is no notable difference in the NKG2A expression between the Rapa group and Rapa+Elt group. In peripheral regulatory and functional NK cells, the AA group demonstrated the higher expression of NKG2D, compared with the NC group (*P* < 0.05). The expression of NKG2D on peripheral regulatory and functional NK cells was upregulated in the CsA group, Rapa group, and Rapa+Elt group compared with the AA group, but there is statistical significance only for functional NK cells (*P* < 0.05). Moreover, there is no notable difference in the NKG2D expression between the Rapa group and Rapa+Elt group. There was no significant difference between the effect of Rapa and CsA on the function of NK cells (Figure 4).

### 3.4. Efficacy of Rapamycin Combined with Eltrombopag on mDC

**Figure 5:**
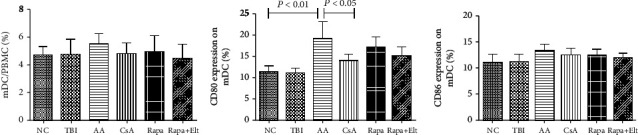
Efficacy of rapamycin combined with eltrombopag on mDC. The numbers of mDC and the expression of CD80 and CD86 on mDC in the CsA group, the Rapa group, and the Rapa+Elt group are all lower than that in the AA group but with no statistical significance except CD80. And the expression of CD80 on mDC was downregulated in the CsA group, the Rapa group compared with the AA group, but there is statistical significance only in the CsA group (*P* < 0.05). There was no significant difference between the Rapa and CsA groups on the function of mDC. When Rapa is combined with Elt, the expression of CD80 and CD86 are downregulated more compared to the Rapa group, but there is no statistical significance.

A previous study reported that mDC is increased in AA patients. We also found that mDC in the AA group outnumbered that of the NC group but had no statistical significance (*P* > 0.05). The numbers of mDC in the CsA group, Rapa group, and Rapa+Elt group are all lower than that in the AA group but with no statistical significance. The expression of CD80 and CD86 on mDC in the AA group was higher than the NC group, but there is statistical significance only about CD80 (*P* < 0.05). And the expression of CD80 on mDC was downregulated in the CsA group and Rapa group, compared with the AA group, but there is a statistical significance only in the CsA group (*P* < 0.05). When Rapa is combined with Elt, the expression of CD80 and CD86 is downregulated more compared to the Rapa group, but there is no statistical significance. Although these results suggested that Rapa+Elt had no statistically significant effect on the numbers of mDC and expression of its functional molecule CD80 and CD86, the combined therapy still indicated that there is a potential synergy with immunosuppressant on AA mice to improve its outcome (Figure 5).

### 3.5. Efficacy of Rapamycin Combined with Eltrombopag on T Lymphocyte Subsets

**Figure 6:**
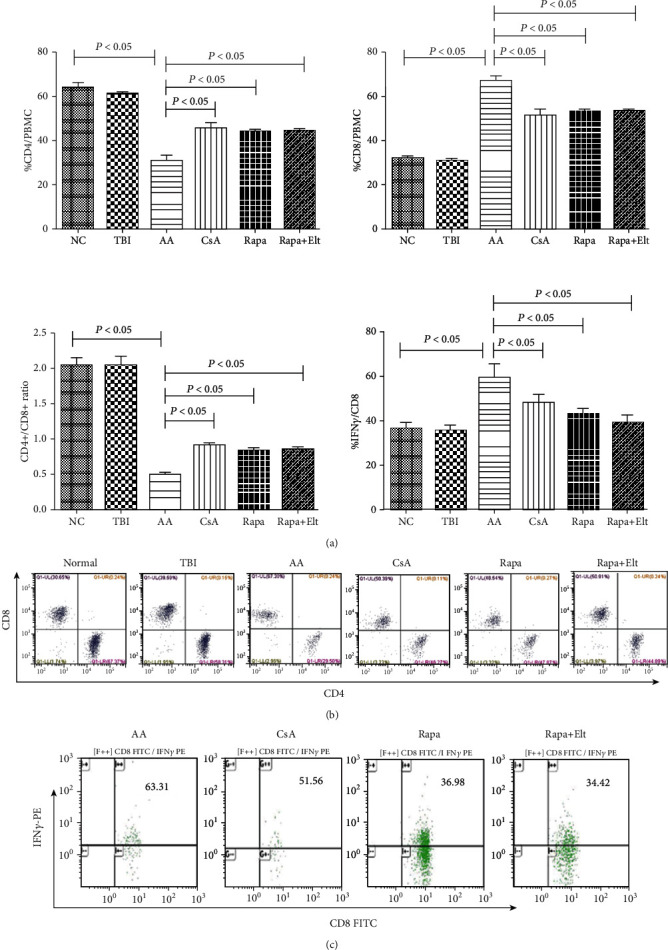
Efficacy of rapamycin combined with eltrombopag on T lymphocytes subsets. (a) Column diagram of the efficacy of rapamycin combined with eltrombopag on CD4+/CD8+T ratio and the secretion IFN-*γ* in CD8+T cells. Compared with the NC group, the AA group showed a decreased CD4+/CD8+ ratio (*P* < 0.05). The results showed that the CD4+/CD8+ ratio in the CsA group, Rapa group, and Rapa + Elt group had an obvious elevation than the AA group (all *P* < 0.05). There was no significant difference among the effect of Rapa, CsA, and Rapa+Elt on the CD4+/CD8+ ratio. As for IFN-*γ*, Rapa can reduce the secretion of IFN-*γ* from CD8+T cells with efficacy similar to that of the standard dose of CsA and had a better outcome when combined with Elt in bone marrow failure mice. (b) Representative flow cytometry analyses of CD4+/CD8+T ratio. (c) Representative flow cytometry analyses of the secretion IFN-*γ* in CD8+T cells.

The CD4+ T cell ratio in the AA group was lower than that in the NC group (*P* < 0.05). The CD4+ T cell ratio in the CsA group, Rapa group, and Rapa+Elt group were notably increased than the AA group (all *P* < 0.05). The CD48 T cell ratio in the AA group was higher than that in the NC group (*P* < 0.05). The CD8+ T cell ratio in the CsA group, Rapa group, and Rapa + Elt group were notably decreased than the AA group (all *P* < 0.05). Compared with the NC group, the AA group showed a decreased CD4+/CD8+ ratio (*P* < 0.05). The results showed that the CD4+/CD8+ ratio in the CsA group, Rapa group, and Rapa+Elt group had an obvious elevation than the AA group (all *P* < 0.05). There were no significant difference among effect of Rapa, CsA, and Rapa+Elt on the CD4+/CD8+ ratio (Figures 6(a) and 6(b)). As for INF-gamma, Rapa can reduce the secretion of IFN-*γ* from CD8+T cells with efficacy similar to that of the standard dose of CsA and had a better outcome when combined with Elt in bone marrow failure mice (Figures 6(a) and 6(c)).

### 3.6. Efficacy of Rapamycin Combined with Eltrombopag on regulatory T Cells

**Figure 7:**
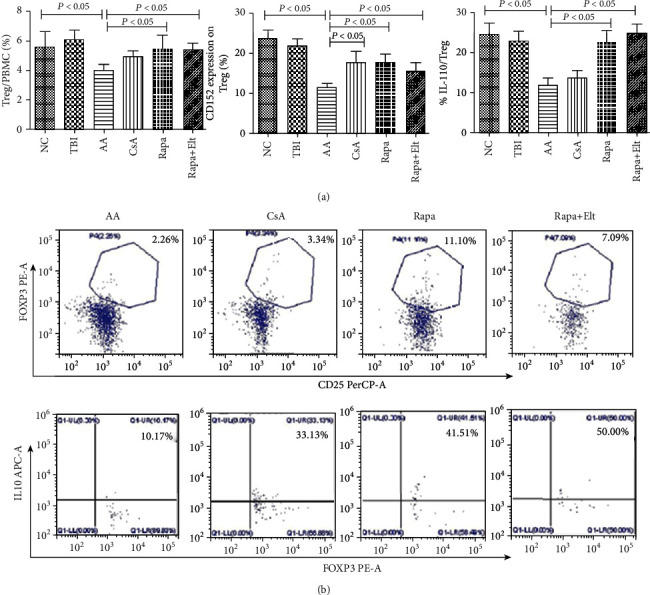
Efficacy of rapamycin combined with eltrombopag on regulatory T cells. (a) The ratio of regulatory T cells increased notably in the AA group after receiving CsA, Rapa, or Rapa+Elt, respectively, and there was a significant difference between the Rapa group and AA group, as well as the Rapa+Elt group and AA group (*P* < 0.05). There was no significant difference between the effect on numbers of Tregs of the Rapa and Rapa+Elt groups. No significant upregulation of the CD152 expression was observed on Tregs in the AA group after receiving Rapa or Rapa+Elt, respectively. As for IL-10, Rapa can increase the secretion of IL-10 from Treg cells compared with the CsA group and had a better outcome when combined with Elt in AA mice. (b) Representative flow cytometry analyses of the secretion IL-10 in Tregs and Tregs/PBMNC ratio after Rapa alone and combined with Elt treatment.

In the AA group, the ratio of the Tregs decreased compared with NC group (*P* < 0.05). The CsA group, Elt group, Rapa group, Rapa + Elt group showed a notable increased ratio of Tregs compared with the AA group, among which there were only the Rapa group and Rapa+Elt group which showed statistical significance (*P* < 0.05). However, there was no significant difference between effect on numbers of Tregs of the Rapa and Rapa+Elt group. (Figures 7(a) and 7(b)).

Furthermore, to determine the effect of Rapa+Elt on Tregs function, we tested the CD152 and IL-10 on Tregs. In the AA group showed decreased expression of CD152 in Tregs than NC group (*P* < 0.05), which is in accordance with previous study. Also, CD152 expression in CsA group, Rapa group, Rapa + Elt group were significantly increased compared with AA group (all *P* <0.05). There was no significant upregulation of CD152 on Treg between Rapa alone and the combined with Elt group. As for IL-10, Rapa can increase the secretion of IL-10 from Treg cells compared with CsA group, and had a better outcome when combined with Elt in bone marrow failure mice. We did not observe the difference of the curative effect of Rapa group and CsA group, but for IL-10/Tregs ratio, Rapa group and Rapa +Elt group were superior to than CsA group (*P* <0.05) (Figures 7(a), 7(b)).

### 3.7. Changes of Plasma Cytokines

**Figure 8:**
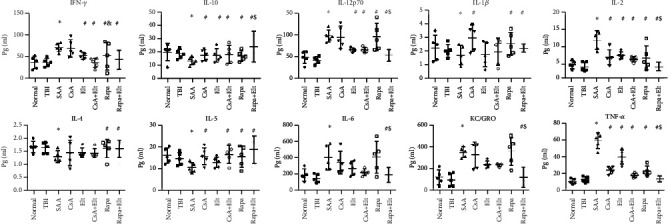
Change of level of plasma cytokines in AA mice after the treatment of rapamycin combined with eltrombopag. ^∗^Compared with the NC group (*P* < 0.05). ^#^Compared with the AA group (*P* < 0.05). ^&^Compared with the CsA group (*P* < 0.05). ^$^Compared with the Rapa group (*P* < 0.05).

We used MSD to determine the cytokine level in different groups. The level of plasma IFN-*γ*, IL-12p70, IL-2, IL-6, KC/GRO, and TNF-*α* in SAA mice was higher than that of the NC group (all *P* < 0.05). In addition, the level of plasma IL-10, IL-1*β*, IL-4, and IL-5 was lower than that of the NC group, but there were only IL-10 and IL-4 which showed statistical significance (*P* < 0.05). Compared with the AA group, the level of plasma IL-2 and TNF-*α* decreased significantly (*P* < 0.05), but IL-10, IL-5, and IL-1*β* increased significantly in the CsA group (*P* < 0.05). Compared with the AA group, the level of plasma IFN-*γ*, IL-2, and TNF-*α* decreased significantly (*P* < 0.05), but IL-10, IL-4, IL-5, and IL-1*β* increased significantly in the Rapa group (*P* < 0.05). There was no significant difference between the CsA and Rapa groups. Although the levels of cytokines of AA mice had shown a certain degree of change after the Elt treatment, the effect of the single treatment was not as good as that of the combined treatment. However, the Rapa+Elt group and Rapa group showed a lower level of IFN-*γ* compared with the CsA group, but there was only a significant difference in the Rapa+Elt group (*P* < 0.05). As for IL-10, IL-12p70, IL-2, IL-6, KC/GRO, and TNF-*α*, the Rapa+Elt group showed a more significant effect than Rapa or Elt alone (Figure 8). Thus, Rapa+Elt significantly downregulated cytokines related to Th1 immune responses, such as IFN-*γ*, and upregulated cytokines related to Th2 immune responses, such as IL-10. To some extent, Rapa combined with Elt has a synergistic effect with CsA and Rapa alone in AA treatment.

### 3.8. The Survival Rate of AA Mice Improved after the Treatment of Rapamycin Combined with Eltrombopag

**Figure 9:**
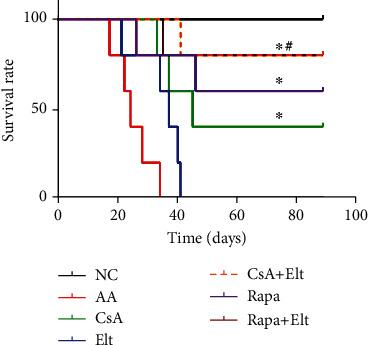
The survival rate of AA mice improved after the treatment of rapamycin combined with eltrombopag. ^∗^Compared with the AA group (*P* < 0.05). ^#^Compared with the Rapa group (*P* < 0.05).

We fed the mice for 90 days and observed the survival period. The results showed that all the mice in the NC group survived for 90 days, all the mice in the AA group died at 90 days, and the 90-day mortality rate of the CsA group, Elt group, CsA+Elt group, Rapa group, and Rapa+Elt group were 60%, 100%, 20%, 40%, and 20%, respectively. As for the survival rate, the CsA group, CsA+Elt group, Rapa group, and Rapa+Elt group showed significantly longer survival time compared with the AA group (*P* < 0.01). There was no significant change in the survival time of mice in the Elt group compared with AA group (*P* > 0.05). The survival of mice of the Rapa+Elt group was significantly higher than that in the Rapa and CsA group (*P* < 0.01) (Figure 9).

## 4. Discussions

The immune system plays an important role in the pathogenesis of AA, which is now recognized as an autoimmune disease. The accumulation of data supporting an immune pathogenesis along with large prospective trials defining the success of IST in SAA formed the rationale for this development [15]. Rapamycin (Rapa) is a kind of macrolide antibiotic with similar structure to FK-506. Rapa was first used as immunosuppressant in organ transplantation, and then, it was used more and more in the treatment of autoimmune diseases. At present, Rapa has been reported to have a good effect in animal experiments [8, 16, 17], which can ameliorate BM failure and improve the immune abnormality of BMF mice. In our study, we demonstrate that rapamycin effectively and reproducibly attenuated in AA mouse models, with efficacy similar to that of the standard dose of CsA. Despite a strong theoretical rationale for the use of Rapa in AA patients, it has not achieved a satisfactory effect in the treatment of SAA patients [9].

It is unclear why Rapa did not yield an improvement in the outcomes in SAA patients compared to those achieved by standard IST in past studies. Therefore, whether Rapa combined with other treatments can improve the efficacy of SAA patients remains further explore.

Eltrombopag is a peptide, small molecule, oral thrombopoietin receptor agonist; it is currently approved as a single or combined agent in patients with AA. The immune and nonimmune properties of Elt may be complementary or synergize with those of immunosuppressive therapies, contributing to a more rapid and robust recovery of blood counts when given in combination in SAA. “Real-world” data thus far have confirmed the effectiveness of Elt in the earlier clinical trials in SAA [18–20].

Apart from erythropoietin and G-CSF, TPO has distinct properties that could be effective in stimulating hematopoietic stem cells (HSCs). However, previous research reports that the endogenous TPO serum levels in SAA patients were very elevated which raised concerns of the ineffectiveness of TPO therapy [21], but the action site of TPO-R of Elt is different from the TPO. It has been reported that Elt plays an important immunomodulatory role in the treatment of immune thrombocytopenic purpura (ITP), such as the decreased release of IFN-*γ* and improved immune balance [22–24]. Moreover, in recent years, it has been discovered that Elt can also mobilize intracellular iron [25].

Whether Rapa combined with Elt can achieve a better curative effect in SAA patients is not clear at present. In our study, we tested the efficacy of Rapa combined with Elt as a new treatment in mouse models of immune-mediated bone marrow failure, whether a better curative effect can be achieved compared to Rapa alone. In this study, although Rapa combined with Elt had no significant improvement effect on the number and function of NK cells and their subsets, mDCs, and CD4+/CD8+ ratio in AA mice compared with Rapa alone, the Rapa+Elt can increase the secretion of IL-10 of Tregs and the number of Tregs, but has no significant effect on the number of Treg cells compared to with Rapa alone. We did not observe the difference in the curative effect of the Rapa group and the CsA group, but for the IL-10/Tregs ratio, the Rapa group was superior to the CsA group. Although Rapa combined with Elt can more reduce IFN-r secretion of CD8+T cells compared to Rapa alone, there was no statistical difference. Compared with the AA group, the level of plasma IFN-*γ*, IL-2, and TNF-*α* decreased significantly (*P* < 0.05), but IL-10, IL-4, IL-5, and IL-1*β* increased significantly in the Rapa group (*P* < 0.05). As for IL-10, IL-12p70, IL-2, IL-6, KC/GRO, and TNF-*α*, the Rapa+Elt group showed a more significant effect than Rapa alone. Additionally, the intervention treatment with Rapa in combination Elt in the AA mouse model more obviously ameliorated pancytopenia and extended animal survival in a manner comparable to the standard dose of CsA and Rapa alone.

Rapa or Elt is currently approved as a single agent in patients with an insufficient response to the initial immunosuppression. Our finding of the immunomodulation function of Rapa+Elt suggests that combination therapy influences the immune balance of AA mice and improves its outcome. In the current study, we found Rapa+Elt plays a significant role in aplastic anemia treatment. Thus, Rapa+Elt significantly downregulated cytokines related to Th1 immune responses and upregulated cytokines related to Th2 immune responses. To some extent, Rapa combined with Elt has a synergistic effect with CsA and Rapa alone in AA treatment. Combination therapy supports the potential clinical utility in aplastic anemia treatment, which may further improve the efficacy of AA patients.

## Figures and Tables

**Table 1:** Analysis of blood cell count of mice in each group on the 17th day (^−^X ± SD).

	n	WBC (×10^9^/l)	RBC (×10^12^/l)	Hb (g/l)	PLT (×10^9^/l)
NC	5	4.10 ± 0.40	10.38 ± 0.22	150.60 ± 8.20	509.60 ± 41.83
TBI	5	3.35 ± 0.61	9.91 ± 0.33	140.4 ± 5.18	440.00 ± 29.62
AA	5	1.10 ± 0.40^#^	7.99 ± 0.52^#^	98.20 ± 9.42^#^	109.60 ± 41.82^#^
CsA	5	2.73 ± 0.62^∗^	8.82 ± 0.40^∗^	119.00 ± 14.37^∗^	328.00 ± 73.30^∗^
Elt	5	1.66 ± 0.41^∗^	8.32 ± 0.31^∗^	126.20 ± 4.76^∗^	288.40 ± 56.53^∗^
CsA+Elt	5	2.85 ± 0.61^∗^	9.43 ± 0.38^∗^	139.40 ± 6.35^∗^	392.00 ± 54.48^∗^
Rapa	5	2.44 ± 0.84^∗^	9.38 ± 0.35^∗^	146.20 ± 7.80^∗^	439.00 ± 27.26^∗^
Rapa+Elt	5	3.55 ± 0.51^∗^	9.46 ± 0.38^∗^	151.20 ± 7.98^∗^	457.74 ± 20.96^∗^

NC: normal control; TBI: total body irradiation; AA: aplastic anemia; CsA: CsA-treated mice; Elt: Elt-treated mice; CsA+Elt: CsA combined with Elt treated mice; Rapa: Rapa-treated mice; Rapa+Elt: Rapa combined with Elt treated mice; ^∗^Compared with the AA group (*P* < 0.01); ^#^Compared with the NC group(*P* < 0.05).

## Data Availability

The data used to support the findings of this study are available from the corresponding author upon request.
